# NRAS mutant melanoma – undrugable?

**DOI:** 10.18632/oncotarget.970

**Published:** 2013-04-13

**Authors:** Christian Posch, Susana Ortiz-Urda

**Affiliations:** University of California San Francisco, Department for Dermatology, Mt. Zion Cancer Research Center, California, USA; University of California San Francisco, Department for Dermatology, Mt. Zion Cancer Research Center, California, USA

Mutations in the three rat sarcoma (RAS) family members NRAS (neuroblastoma-RAS), HRAS (Harvey-RAS) and KRAS (Kirsten-RAS) are found in one third of human cancers. Among the first oncogenes discovered in cutaneous melanoma was NRAS, which is mutant in up to 20% of tumors causing aberrant signaling in several downstream cascades. Despite, being a highly relevant therapeutic target, design of small molecules selectively inhibiting mutant NRAS in melanoma, to date, remains an unsolved challenge. The end?

RAS proteins are molecular switches mediating signals from ligand activated receptor tyrosine kinases (RTK) to the nucleus through a complex network of downstream signaling cascades. Although NRAS, KRAS and HRAS share structural and functional similarities, recent findings suggest distinctive subcellular localization and compartmentalized signalling of these isoforms. This is thought to contribute to differences in protein function in the RAS family, but may also explain signaling variations and predominance of certain RAS mutations across different cancer types. Whereas KRAS mutations are frequent in colorectal cancer, lung cancer and pancreatic cancer, NRAS mutations are by far the predominant alteration among RAS isoforms in melanoma. The majority of NRAS mutations are found in codon 61 impairing the enzymatic activity of RAS to cleave GTP to GDP. Other, less frequent mutations are found in codon 12 and 13 preventing the association of GAPase activating proteins (GAP), which accelerate the weak hydrolytic potential of RAS. As a result, NRAS remains in its active, GTP-bound state driving cell proliferation, survival and motility making NRAS an important therapeutic target in melanoma. What are the challenges in designing effective inhibitors of mutant RAS?

So far, several different strategies of directly targeting RAS have not resulted in effective therapeutics. One approach is based on the concept of inhibiting the association of RAS with GTP. Unlike kinases, where ATP binds and activates at low micromolar concentrations, the affinity for GTP to RAS is in the low picomolar range making the development of specific GTP antagonists to date impossible. A second approach is based on restoring the enzymatic activity of mutant RAS with GAP-like molecules that enhance RAS-GAP association and promote cleavage of GTP. So far, endeavours to directly inhibit RAS have not translated into a clinical success, thus, the central focus of research efforts has become indirect inhibition of RAS. This entails a growing understanding of essential post translational modifications (farnesylation), the membrane association and the complex downstream signaling network of RAS. Farnesylation is a lipid modification necessary for RAS function. Several farnesylate inhibitors (FTI) entered clinical studies, but failed to confirm the high pre-clinical, anti tumor activity. Although FTIs potently block farnesylation in HRAS models, an unexpected biochemical difference among the RAS isoforms revealed alternative posttranslational modifications that can substitute farnesylation, largely limiting the use of FTIs as anti-RAS therapeutics. The localization of RAS to the plasma membrane is also critical for the interaction of RAS with various downstream effectors. It is hoped that interference with docking proteins such as prenyl binding sites on the plasma membrane may help to prevent stimulus independent signaling by mutant RAS.

**Figure 1:**
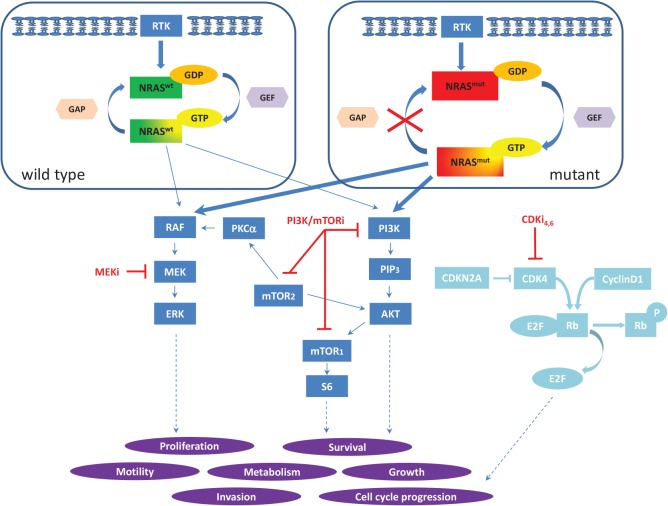
Model of NRAS signaling in melanoma Wild type NRAS (top left) cycles between an inactive GDP-bound and active GTP-bound state, whereas mutations in G12, G13 and Q61 prevent hydrolysis of GTP, locking mutant NRAS in its GTP-bound, active state (top right) which results in permanent, stimulus independent downstream signaling. NRAS-GTP activates downstream effectors of the MAPK and PI3K/mTOR pathway (dark blue boxes). Schematic of the cell cycle pathway (light blue) and interaction sites of the specific inhibitors in the different cascades (red lines). (RTK: receptor tyrosine kinases, GAP: GAPase activating protein, GEF: guanosine exchange factor, MEKi: MEK inhibitor, PI3K/mTORi: PI3K/mTOR inhibitor, CDKi_4,6_: CDK_4,6_ inhibitor)

Recently, the focus of indirect RAS inhibition has shifted to interfere with the complex network of activated downstream cascades such as the mitogen activated protein kinase (MAPK), phosphoinsitol 3-kinase (PI3K), phospholipid C (PLC), RAL and the cell cycle pathway among others (Figure 1). Although a recent clinical trial with MEK162, a potent MEK inhibitor, has shown some activity in patients with NRAS mutant melanoma (Ascierto PA et al.), innate or acquired tumor resistance to single-targeted agents is inevitable. However, there is reasonable hope that the concept of combined selective pathway inhibition may be effective. Our group has recently demonstrated the importance of MAPK and PI3K/mTOR signaling in a large collection of primary and metastatic, patient derived melanoma samples as well as in 10 human NRAS mutant melanoma cell lines (Posch C et al.). Blocking with specific inhibitors in these two pathways synergistically decreased cell viability *in vitro* and regressed NRAS mutant xenografts *in vivo*. It is important to notice that only a certain ratio of the MEK to the PI3K/mTOR inhibitor showed synergism across all 10 cell lines. Although a direct line between preclinical results and applications *in vivo* cannot be drawn, this finding suggests that the most effective balance of two drugs might not be at the maximum tolerated concentration of those drugs *in vivo*.

Another group discovered CDK4 as a coextinction target with MEK in NRAS mutant melanoma (Kwong LN et al). Based on an elegant, mainly computational analysis of large data sets they found that the combination of a selective MEK and CDK_4,6_ inhibitor regressed tumors of two independent NRAS mutant cell lines in a mouse xenograft model. However, the constitutive CDKN2A knockout mouse model used in this study and the fact, that alterations in the cell cycle pathway are common genetic events in human melanoma, make it unlikely that NRAS status alone is a marker for effective therapy with MEK+CDK_4,6_ inhibition. For successful translation into clinics it will be essential to test more NRAS mutant cell lines for the activity of inhibitor combinations and to refine and fully characterize the genetic profile of melanoma cells that are most likely to respond.

Both discussed combinations for pathway interference (MEK+PI3K/mTOR and MEK+CDK_4,6_) are not tumor specific therapies and bear the risk of severe side effects, as most cell types and tissues signal through these pathways and will also be affected. Still, the concept of oncogene addiction and over-activation of certain cascades allows for some selectivity to inhibit mainly tumor cells. It is to date the most promising strategy to interfere with currently undrugable targets such as mutant NRAS in melanoma.

